# Effect of exercise training on cardiac mitochondrial respiration, biogenesis, dynamics, and mitophagy in ischemic heart disease

**DOI:** 10.3389/fcvm.2022.949744

**Published:** 2022-10-11

**Authors:** Mary Audrey D. Viloria, Qing Li, Wang Lu, Nguyen Thanh Nhu, Yijie Liu, Zhen-Yang Cui, Yu-Jung Cheng, Shin-Da Lee

**Affiliations:** ^1^Department of Physical Therapy, Graduate Institute of Rehabilitation Science, China Medical University, Taichung, Taiwan; ^2^Department of Physical Therapy, College of Health Sciences, Mariano Marcos State University, Batac, Philippines; ^3^Department of Rehabilitation, Shanghai Xuhui Central Hospital, Shanghai, China; ^4^Department of Traditional Treatment, Longhua Hospital, Shanghai University of Traditional Chinese Medicine, Shanghai, China; ^5^Faculty of Medicine, Can Tho University of Medicine and Pharmacy, Can Tho, Vietnam; ^6^School of Rehabilitation Medicine, Shanghai University of Traditional Medicine, Shanghai, China; ^7^Institute of Rehabilitation Medicine, Shanghai University of Traditional Medicine, Shanghai, China; ^8^School of Rehabilitation Medicine, Weifang Medical University, Weifang, China; ^9^Department of Physical Therapy, Asia University, Taichung, Taiwan

**Keywords:** ischemic heart disease, exercise training, mitochondria, cardioprotection, intervention

## Abstract

**Objective:**

Cardiac mitochondrial dysfunction was found in ischemic heart disease (IHD). Hence, this study determined the effects of exercise training (ET) on cardiac mitochondrial respiration and cardiac mitochondrial quality control in IHD.

**Methods:**

A narrative synthesis was conducted after searching animal studies written in English in three databases (PubMed, Web of Science, and EMBASE) until December 2020. Studies that used aerobic exercise as an intervention for at least 3 weeks and had at least normal, negative (sedentary IHD), and positive (exercise-trained IHD) groups were included. The CAMARADES checklist was used to check the quality of the included studies.

**Results:**

The 10 included studies (CAMARADES score: 6–7/10) used swimming or treadmill exercise for 3–8 weeks. Seven studies showed that ET ameliorated cardiac mitochondrial respiratory function as manifested by decreased reactive oxygen species (ROS) production and increased complexes I-V activity, superoxide dismutase 2 (SOD2), respiratory control ratio (RCR), NADH dehydrogenase subunits 1 and 6 (ND1/6), Cytochrome B (CytB), and adenosine triphosphate (ATP) production. Ten studies showed that ET improved cardiac mitochondrial quality control in IHD as manifested by enhanced and/or controlled mitochondrial biogenesis, dynamics, and mitophagy. Four other studies showed that ET resulted in better cardiac mitochondrial physiological characteristics.

**Conclusion:**

Exercise training could improve cardiac mitochondrial functions, including respiration, biogenesis, dynamics, and mitophagy in IHD.

**Systematic review registration:**

https://www.crd.york.ac.uk/prospero/ display_record.php?RecordID=226817, identifier: CRD42021226817.

## Introduction

Ischemic heart disease (IHD) is caused by oxygen deprivation in the heart secondary to blockage in the arteries supplying oxygen-rich blood to the heart (1), which results in heart attack, also known as myocardial infarction (MI), leading to increased prevalence of morbidity and mortality (2).

Mitochondria are essential to survival, as it sustains cellular function through adenosine triphosphate (ATP) synthesis (3). In IHD, the heart does not receive enough ATP due to cardiac mitochondrial dysfunction (3, 4). This ATP deficiency is usually the result of decrease in electron transport chain (ETC) complex activity, expression of complex I sub-units, respiratory control ratio (RCR), and superoxide dismutase 2 (SOD2). Moreover, the increase in reactive oxygen species (ROS) production that results in oxidative stress also contributes to the decrease in ATP production (3–7).

Emerging evidence showed that mitochondrial quality control (MQC), which includes mitochondrial biogenesis, dynamics, and mitophagy, are dysregulated in IHD. Particularly, different proteins and enzymes involved in the mitochondrial biogenesis, such as the peroxisome proliferator-activated receptor gamma coactivator 1-alpha (PGC-1α), phosphoinositide 3-kinase (PI3K), protein kinase B (AKT) nuclear respiratory factor 1 and 2 (NRF 1/2), mitochondrial transcription factor A (TFAM), sirtuin 1 and 3 (SIRT 1/3), single-stranded DNA Protein (SSBP1), DNA polymerase gamma gene (POLG/PLOG), and DNA topoisomerase I mitochondrial (TOP1MT), are decreased in IHD, which causes dysregulated cardiac mitochondrial biogenesis (6–10). As a consequence of oxidative stress, the expression of the energy-sensing enzyme, AMP-activated protein kinase (AMPK), is heightened in IHD (11). Regarding the biomarkers for MQC, a significant disproportion between the mitochondrial fusion proteins, such as mitofusin 1 (MFN1), mitofusin 2 (MFN2), optic atrophy 1 (OPA1), and mitochondrial fission proteins, such as dynamin-related protein 1 (DRP1), was observed in IHD (12). Additionally, different studies showed downregulation of different mitophagy biomarkers, such as the PTEN-induced kinase 1 (PINK), Parkin, and suquetosome 1 (P62) (13–15), while the autophagic biomarkers, such as the microtubule-associated protein light chains 1 and 3 (LC3II/I), and Beclin 1, are notably increased in IHD, and that such expressions were reported to have detrimental effects on the condition (14, 16).

Exercise training (ET) has been accepted as a safe technique in cardiac rehabilitation. In fact, it has been considered to cause both primary and secondary prevention of IHD. As an effect, different studies reported an overall reduction in the prevalence of morbidity and mortality secondary to IHD (17, 18). In relation to cardiac mitochondrial function, previous studies reported that ET increases tolerance to decreased oxygen, which leads to an improvement in mitochondrial respiration (19, 20). Moreover, studies that investigated the effects of ET on the different mechanisms involved in MQC have shown an improvement in MQC as a whole (21, 22).

However, despite the numerous studies that investigated on the effects of ET on the mitochondrial function in IHD, no systematic review has been conducted regarding this issue. Due to the complexity of the mechanisms involved in cardiac mitochondrial function, as well as with the contrasting findings about what really transpire pre-and/or-post ET in pre-and/or-post IHD, this makes the topic being poorly understood. Therefore, this systematic review was conducted to understand the effects of ET on cardiac mitochondrial function in IHD, specifically on the cardiac mitochondrial respiration, MQC, and mitochondrial physiological characteristics.

## Materials and methods

### Protocol and registration

This systematic review is registered in the International Prospective Register of Systematic Review (PROSPERO) with PROSPERO ID number CRD42021226817. The report followed the Preferred Reporting Items for Systematic Reviews and Meta-Analyses (PRISMA) checklist and the PRISMA 2020 abstract checklist to ensure the quality of this systematic review (23).

### Eligibility criteria

#### Study design type

Controlled trial animal studies written in English, without restrictions on the publication date, were used in this systematic review. We excluded protocol articles, reviews, abstracts, and case reports.

#### Animal model type

Studies that used any animal species induced with IHD regardless, but with specification, of gender, age, and type of species were included. Moreover, we considered studies that elaborated how and when during the study that IHD was induced.

#### Intervention focus

Studies which utilized any type of aerobic ET (e.g., swimming and treadmill training) for at least 3 weeks regardless of the duration and frequency were included. Other studies that used aerobic ET but with additional treatment such as, but not limited to, resistance exercise and/or medications, were all excluded.

#### Comparators

Studies with at least a normal (sham or not), negative (sedentary IHD), and positive (exercise-trained IHD) groups were included.

#### Outcomes

To understand the effect of ET on the cardiac mitochondrial function in IHD, the cardiac mitochondrial respiration was considered the primary outcome. This included components of the electron ETC, such as complexes I–V activity, cytB, NADH dehydrogenase subunits (ND1 and 6), as well as the production of ROS, SOD2, RCR, and ATP.

The cardiac MCQ was considered the secondary outcome. This included the different biomarkers involved in the following three MQC mechanisms: (1) cardiac mitochondrial biogenesis, such as those comprising different replication and transcription processes on both the mitochondrial and nuclear levels; (2) cardiac mitochondrial dynamics, which included both fission and fusion factors; and (3) mitophagy and autophagosomal factors.

Moreover, the cardiac mitochondrial physiological characteristics were also investigated, which included mitochondrial density, shape, size, and the mitochondrial membrane potential (MMP).

### Information sources

Relevant studies were searched and identified through the PubMed, Web of Science, and EMBASE databases. Additional articles were manually searched from the reference list of the initially searched articles. This was carried out from November to December 2020.

### Search strategy

The search strategy consisted of keywords that included the following three areas of specification: (1) animal population, (2) intervention (aerobic exercise), and (3) any area related to mitochondrial function. Using the Boolean operators, the following terms were combined: (“Ischemic heart disease” OR “Myocardial Infarction” OR “Coronary Artery Disease”) AND (“Exercise Training” OR “Aerobic Exercise” OR “Swimming” OR “Treadmill”) AND (“mitochondria” OR “mitochondrial” OR “mitochondrion” OR “mitochondrial respiration” OR “mitophagy” OR “mitochondrial biogenesis” OR “ATP” OR “SIRT” OR “AMPK” OR “PGC-1α” OR “TFAM” OR “NRF” OR “mitochondrial dynamics” OR “mito-fusion” OR “mito-fission”).

### Selection process

Two independent evaluators screened the titles and abstracts of the initially identified studies for duplication or for non-related topics. Subsequently, the same evaluators thoroughly reviewed the full text of the accepted studies based on the eligibility criteria that were mentioned earlier. A discussion with a third evaluator was done to finalize the decision in case of any disagreement.

### Data records

#### Data management

All studies included in this systematic review are kept on a specific virtual library using EndNote 20. The write-up, tables, and figures are kept in a password-protected Google drive, and are readily available for reference or review purposes.

#### Data collection process

The data about the study characteristics and outcomes in this systematic review were obtained through extensive reading and analysis of texts, graphs, and tables from the methodology and results section of the included studies.

#### Data items

In line with the study characteristics, we included the last name of the first author, the year of study publication, the animal model used (type of species and age), the type of exercise used (frequency and duration included), and the type of IHD (acute ischemia or MI).

For the outcomes, we included results obtained from the cardiac mitochondria of the animals. Specifically, data submitted included the cardiac mitochondrial respiratory function, biogenesis, dynamics (fusion and fission), mitophagy, and physiological characteristics.

Data were based and presented regarding what has been reported in the included studies. Nevertheless, for missing or unclear data, an e-mail was forwarded to corresponding author/authors for clarification. No data were excluded due to non-response of the authors. Instead, two evaluators discussed the findings through further analysis of the results. Regardless, no assumptions were made on missing or unclear data.

### Outcome and prioritization

The main outcome of this systematic review was the cardiac mitochondrial respiratory function. This was chosen, as it involves different processes necessitating oxygen to convert stored energy into ATP, which is needed by the cardiomyocytes for it to function properly. To warrant proper function of the mitochondrial respiration, the different properties of the cardiac MQC were also investigated, making the cardiac mitochondrial biogenesis, dynamics, and mitophagy as the secondary outcomes. Moreover, the cardiac mitochondrial physiological characteristics, which depict the morphological aspect of the cardiac mitochondria, were also included as a secondary outcome.

### Risk of bias assessment

To ensure the quality of this systematic review, two researchers independently evaluated each study, which was included using the Collaborative Approach to Meta-Analysis and Review of Animal Data from Experimental Studies (CAMARADES) checklist, a 10-item checklist that assesses risk of bias in pre-clinical animal studies. In case of disagreement, the evaluators in-charge discussed it with a third evaluator to obtain a final decision.

### Data synthesis

The data collection process was presented using the PRISMA flowchart template. The study characteristics included the last name of the authors of each study, the publication date, the animal model, the type of IHD, and the ET used. The primary and secondary outcomes of the study that summarized the effect of IHD and ET (in the exercise-trained IHD) in the cardiac mitochondrial function were presented through text, tables, and figure, with increase (↑) and decrease (↓) as the measuring variables, and also presented in frequency (number of studies which presented an outcome as increased or decreased) for each outcome (in text).

## Results

### Search results

Initial search included 203 articles from PubMed (*n* = 84), Web of Science (*n* = 60), and EMBASE (*n* = 59). Four articles were found through the reference lists. After removing duplicates (*n* = 67), the title and abstract of 140 articles were reviewed, through which the irrelevant studies were identified (*n* = 96).

**Figure 1:**
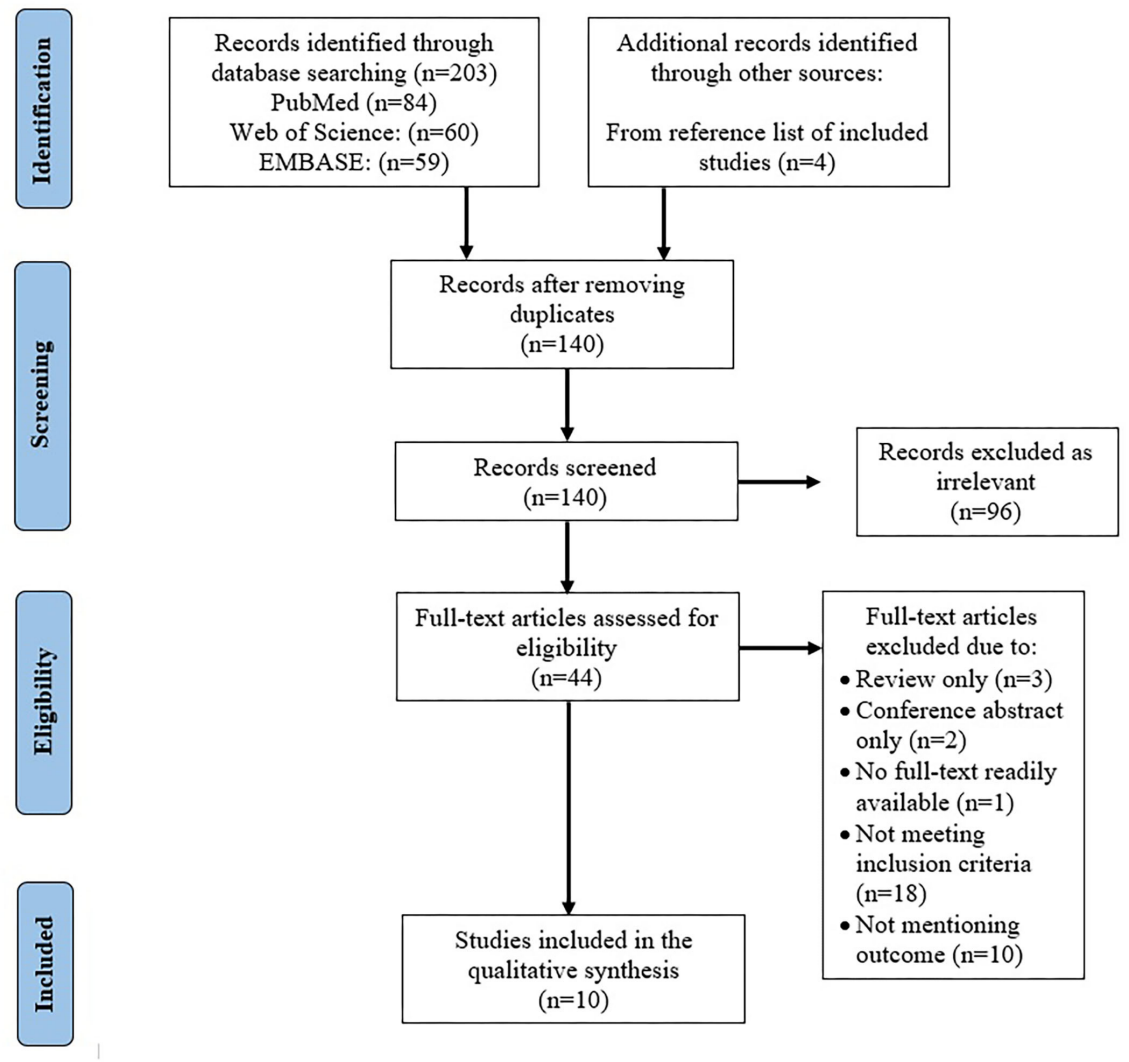
Data flow.

A total of 44 articles underwent full-text review wherein 31 more articles were removed secondary to the following reasons: review articles (*n* = 3), conference abstracts (*n* = 4), studies that were not able to meet the inclusion criteria, such as duration of ET and undergoing additional treatment, non-RCT, non-IHD articles, human studies (n=18), and those that did not mention any of the outcomes of the systematic review conducted (*n* = 10). After the thorough review, a total number of 10 articles were included in this systematic review (Figure 1), with CAMARADES score ranging from 6 to 7/10 (Table 1).

**Table 1:** The quality of included studies based on CAMARADES checklist.

**References**	**CAMARADES Checklist of Study Quality**										
	**1**	**2**	**3**	**4**	**5**	**6**	**7**	**8**	**9**	**10**	**Total**
1. Kraljevic et al. (24)	✓		✓			✓	✓		✓	✓	**6**
2. Jiang et al. (25)	✓	✓	✓			✓	✓		✓		**6**
3. Jiang et al. (26)	✓	✓	✓			✓	✓		✓	✓	**7**
4. Tao et al. (27)	✓	✓				✓	✓		✓	✓	**6**
5. Lu et al. (28)	✓	✓	✓			✓	✓		✓		**6**
6. Ghahremani et al. (29)	✓	✓	✓			✓	✓		✓	✓	**7**
7. Ebadi and Damirchi (30)	✓		✓			✓	✓		✓	✓	**6**
8. Zhao et al. (31)	✓		✓			✓	✓		✓	✓	**6**
9. Ghahremani et al. (32)	✓	✓	✓			✓	✓		✓	✓	**7**
10. Jia et al. (33)	✓	✓	✓			✓	✓		✓	✓	**7**

1. Publication in peer-reviewed journal, 2. Statement of control of temperature, 3. Randomization of treatment or control, 4. Allocation concealment, 5. Blinded assessment of outcome, 6. Avoidance of anesthetics with marked intrinsic properties, 7. Use of animals with IHD, 8. sample size calculation, 9. Statement of compliance with regulatory requirements, 10. Statement regarding possible conflict of interest, ✓: Yes.

### Study characteristics

#### Type of IHD models

A total of eight studies used rats, while two studies used mice. Specifically, five studies used Sprague-Dawley rats, three studies used Wistar rats, and two studies used C57BL/6 (J) mice as models of IHD. The animal models were 8 weeks to 18 months old. One study, however, failed to provide the specific age of the animal model, but described them as adult, instead.

The animal models that underwent surgery were anesthetized using sodium pentobarbital, sodium thiopental, isoflurane, or ketamine with a mixture of either xylazine or sevoflurane, prior to the ligation of the left coronary artery (LCA) to induce IHD (MI or IR). On the one hand, eight studies used MI as a form of IHD, wherein models on seven studies exhibited chronic MI, while one study presented acute MI. On the other hand, two studies used IR as a form of IHD, where ischemia was induced *via* occlusion of the LCA for 30 min followed by reperfusion for 90 min. The types of IHD models used by the included studies are summarized in Table 2.

**Table 2:** Effects of ischemia and exercise training on the cardiac mitochondrial physiological characteristics (MPC) and cardiac mitochondrial respiratory function.

**References**	**Model**	**Exercise type**	**Ischemic type**	**Outcomes**				
				**Mitochondrial physiological characteristics**	**Mitochondrial respiratory functions/ ROS**	**Mitochondrial biogenesis**	**Mitochondrial dynamics**	**Mitophagy**
1. Kraljevic et al. (24)	Sprague-Dawley Female Rats (*adult*)	E: Treadmill D: 70 mins x 5 days/week x 8 weeks	Chronic Myocardial Infarction		**MI:** RCR was reduced using complex I substrate while remained the same as control group using complex II substrate; Decreased complex I, II, III and V activity; Decreased ATP production using both complex I while it increased using complex II substrates. **ET:** Increased RCR using both complex I and II substrates; Increased complexes I, II, and V activity; decreased complex III; Increased ATP production using both complexes I and II substrates.	**MI:** Increased PGC1α **ET:** Decreased PGC1α		
2. Jiang et al. (25)	Sprague-Dawley Male Rats (Age: 8 weeks old)	E: Treadmill D: 1 hr/day x 5 days/week x 8 weeks	Chronic Myocardial Infarction	**MI:** Decreased mitochondrial density **ET:** Increased mitochondrial density	**MI:** Decreased complexes I and III;Decreased SOD 2; Increased ROS **ET:** Increased complexes I and III;Increased SOD 2; Decreased ROS	**MI:** Decreased in PGC-1α, PI3K, Akt, AMPK phosphorylation, SIRT 3 and NRF2 levels **ET:** Increased PGC1α, PI3K, AKT, AMPK, SIRT 3, NRF 2 levels		
3. Jiang et al. (26)	Sprague-Dawley Male Rats (Age: 8 weeks old)	E: Treadmill D: 1 hr/day x 5 days/week x 8 weeks	Chronic Myocardial Infarction	**MI:** Decreased MMP **ET:** Increased MMP	**MI:** Decreased RCR and complexes I-V **ET:** Increased in RCR and complexes I-V	**MI:** Decreased PGC1α and TFAM **ET:** Increased PGC1α levels and expression of TFAM	**MI**: Decreased levels of MFN1, MFN2 and OPA1;Increased DRP1. **ET:** Increased MFN1, MFN2 and OPA1; Decreased DRP1.	
4. Tao et al. (27)	Male C57BL/6 Mice (Age: 10-12 weeks old)	E: Swimming D: 10-90 mins twice daily x 3 weeks	Acute Myocardial Infarction		**MI:** Increased ND1, and ND6 levels; Decreased cytB **ET:** Further increased the levels of ND1 and ND6. Increased cytB.	**MI:** Increased levels of PGC1α, NRF 1&2, TFAM SSBP1, TOP1MT, and POLG. **ET:** Further increased these biomarkers		**MI:** Decreased level of P62; Increased Beclin 1 and LC3II. **ET:** Increased P62; Decreased Beclin 1 and LC3II
5. Lu et al. (28)	Female Sprague Dawley Rats (8-10 weeks old)	E: Treadmill D: 59-60 mins/day x 5 days/week x 8 weeks	Chronic Myocardial Infarction		**MI:** Decreased ATP production **ET:** Increased in ATP production	**MI:** Increased levels of PI3K and AKT; Decreased in the level of AMPK. **ET:** Decreased levels of PI3K and AKT; Increased in the level of AMPK.		
6. Ghahremani et al. (29)	Male Wistar Rats (Age: 8 weeks old)	E: Treadmill D: 15-60 mins/day x 5 days/week x 8 weeks	Acute Ischemia (I: 30 mins; R: 90 mins)				**IR:** Increased MFN1 and DRP1; Decreased Mfn2. **ET:** Increased MFN11 and MFN2 levels; Decreased DRP1 level	
7. Ebadi and Damirchi (30)	Male Wistar Rats (Age: 16 weeks old)	E: Treadmill D: 60 mins/day x 5 days/wk x 6 weeks	Chronic Myocardial Infarction			**MI:** Decreased levels of PGC1α. **ET:** Increased levels of PGC1α.	**MI: D**ecreased protein levels of MFN2; Increased DRP1 **ET:** Increased MFN2 protein levels; Decreased DRP1 protein levels	**MI: D**ecreased levels of Parkin and P62; **ET:** Increased Parkin and P62 levels on moderate and low exercise intensity levels only
8. Zhao et al. (31)	Male C57BL/6J mice (Age: 18 months old)	E: Swimming D: 15 mins or 60 mins/day x 5 days/week x 8 weeks	Chronic Myocardial Infarction	**MI:** Increased mitochondrial size **ET:** Decreased mitochondrial size	**MI:** Decreased SOD2 productionIncreased ROS production. **ET:** Increased SOD2 productionDecreased ROS production.	**MI:** Decreased SIRT3 levels **ET:** Increased levels of SIRT3	**MI:** Increased levels of MFN1, MFN2, OPA1 and DRP 1. **ET:** Both short and long duration exercise decreased MFN1, MFN2 and DRP1.OPA1 in short duration ET increased further while it was decreased in long duration ET.	**MI:** Decreased levels of PINK and Parkin;Increased levels of LC3II and P62 protein. **ET:** Increased levels of PINK and Parkin on both short and long durationDecreased levels of LC3II on both short and long duration ET; Decreased P62 in short duration but increased in long duration ET
9. Ghahremani et al. (32)	Male Wistar Rats (8 weeks old)	E: TreadmillD: 15-60 mins/day x 5 days/wk x 8 weeks	Acute Ischemia(I: 30 mins; R: 90 mins)					**IR:** Increased Beclin 1 expression **ET:** Decreased Beclin 1 levels
10. Jia et al. (33)	Sprague-Dawley Male Rats (Age: 8 weeks old)	E: Treadmill D: 30-60 mins daily x 4 weeks	Chronic Myocardial Infarction	**MI:** Increased number of mitochondria;Decreased mitochondrial size and myocardial MMP. **ET:** Decreased number of mitochondria; Increased mitochondrial size and myocardial MMP.	**MI:** Increased ROSIncreased SOD2; Decreased levels of ND1, ND6, cytB. **ET:** Decreased ROS; Further increased in SOD2; Increased levels of ND1, ND6, cytB.	**MI:** Increased PGC1α and p-PI3K and p-AKT;Decreased SIRT 1;Increased SSBP1; Decreased TOP1MT and POLG **ET:** Further increased PGC1α and p-PI3K and p-AKT; Increased SIRT1; Further increased SSBP1; Increased TOP1MT and POLG		

D, duration of exercise; IR, ischemia-reperfusion compared to normal; MI, myocardial infarction compared to normal; ET, exercise training compared to sedentary IR or MI. (Mitochondrial Characteristics) MMP, mitochondrial membrane potential. (Mitochondrial Respiratory Function) RCR, respiratory control ratio; ATP, adenosine triphosphate; ROS, reactive oxygen species; SOD2, mitochondrial superoxide dismutase; ND1, NADH dehydrogenase 1; ND6, NADH dehydrogenase 6; cytB, cytochrome B. (Mitochondrial Biogenesis) PGC-1α, peroxisome proliferator-activated receptor gamma coactivator 1-Alpha; PI3K, phosphoinositide 3-kinase; AKT, protein kinase B; AMPK, AMP-activated protein kinase; SIRT3, sirtuin 3; NRF 1/2, nuclear respiratory factor 1/2; TFAM, mitochondrial transcription factor A; POLG, DNA polymerase gamma gene; TOP1MT, mitochondrial DNA topoisomerase I; SSBP1, single-stranded DNA binding protein. (Mitochondrial Dynamics) MFN1, mitofusin 1; MNF2, mitofusin 2; OPA1, optic atrophy 1; DRP1, dynamin-related protein 1. (Mitophagy) PINK, PTEN-induced kinase 1; P62, Suquestosome 1; LC3II, Microtubule-Associated Protein Light Chain 3.

#### Type of ET protocol

The included studies used preventative (*n* = 3) or post-IHD (*n* = 7) ET using either treadmill (*n* = 8) or swimming (*n* = 2). These ET sessions were conducted for 3–8 weeks, around 20–180 min/day for 5–7 days a week. Table 2 shows the summary of the ET protocols used.

#### Type of outcomes

For the primary outcome (cardiac mitochondrial respiration), seven studies investigated the different biomarkers involved in this process, such as the levels of ROS (*n* = 3), SOD (*n* = 3), RCR (*n* = 2), ETC complex activity (*n* = 3), ND1 or 6 (*n* = 2), cytB (*n* = 2), and ATP (*n* = 2).

For the secondary outcomes, eight studies assessed the cardiac mitochondrial biogenesis regulators, which included PGC-1α (*n* = 6), PI3K (*n* = 3), AKT (*n* = 3), AMPK (*n* = 2), SIRT3 (*n* = 2), NRF 1/2 (*n* = 1/2), TFAM (*n* = 2), POLG/PLOG (*n* = 1), TOP1MT (*n* = 1), and SSBP1 (*n* = 1). For cardiac mitochondrial dynamics, four studies examined the different levels of the proteins or genes involved in mitochondrial fission, such as the DRP1 (*n* = 4) and mitochondrial fusion, such as the MFN1 (*n* = 3), MFN2 (*n* = 4), and OPA1 (*n* = 2). Regarding mitophagy, four studies investigated the different enzymes and/or proteins involved in the dysfunctional mitochondria, such as PINK1 (*n* = 1), Parkin (*n* = 2), P62 (*n* = 3), LC3II (*n* = 2), and Beclin 1 (*n* = 2).

Finally, four studies analyzed the mitochondrial physiological characteristics such as the mitochondrial number (*n* = 2), density (*n* = 1), and size (*n* = 2), as well as the cardiac MMP (*n* = 2). Table 2 shows the summary of all the outcomes used.

### Outcomes

#### Effects of ET on cardiac mitochondrial respiration in IHD

Three studies (Tables 2, 3) analyzed the effect of IHD on ROS regulation (25, 31, 33). All three studies showed that ROS was increased in IHD and was reversed by ET. The same three studies also investigated the level of the SOD2 in IHD. Of these, two studies showed decreased SOD2 in IHD compared with normal controls (25, 31), while one study reported the opposite (33). Regardless of the contrasting results, ET increased the expression of SOD2 in the three studies (25, 31, 33). Moreover, a report of decreased RCR in two studies was noted in IHD, whereas ET ameliorated it (24, 26).

**Table 3:** Effects of ischemia and exercise training on cardiac mitochondrial respiration and physiological characteristics.

**References**	**Ischemic heart**											**Exercise training**										
	**MPC**			**Mitochondrial respiratory function**								**MPC**			**Mitochondrial respiratory function**							
	**Number/ Density**	**MMP**	**Size**	**ROS**	**SOD2**	**RCR**	*Complex	**ND1**	**ND6**	**CytB**	**ATP**	**Number/ ·Density **	**MMP**	**Size**	**ROS**	**SOD2**	**RCR**	*Complex	**ND1**	**ND6**	**CytB**	**ATP**
Kraljevic et al. (24)						↓/S	↓				↓/ ↑						↑	↑/ ↓				↑
Jiang et al. (25)	·↓			↑	↓		↓					·↑			↓	↑		↑				
Jiang et al. (26)		↓				↓	↓						↑				↑	↑				
Tao et al. (27)								↑	↑	↓									↑↑	↑↑	↑	
Lu et al. (28)											↓											↑
Zhao et al. (31)			↑	↑	↓									↓	↓	↑						
Jia et al. (33)	↑	↓	↓	↑	↑			↓	↓	↓		↓	↑	↑	↓	↑↑			↑	↑	↑	

(Mitochondrial Characteristics) MMP, mitochondrial membrane potential. (Mitochondrial Respiratory Function) RCR, respiratory control ratio; ATP, adenosine triphosphate; ROS, reactive oxygen species; SOD2, mitochondrial superoxide dismutase; ND1, NADH dehydrogenase 1; ND6, NADH dehydrogenase 6; cytB, cytochrome B; (Others) ↑, Increased; ↑↑, further increased; ↓, Decreased; S, Same with control; Red Arrow, effect of ischemia; Blue Arrow, effect of exercise training; ^*^, refer to Table 1.

Three studies reported decreased activity of the five ETC complexes in IHD (24–26), whereas ET enhanced the activity of complexes I (24–26), II and IV (24, 26), and V (26). A decreased complex III activity in the exercise-trained IHD compared with the sedentary IHD was reported in one study (24), which was countered by two other studies (25, 26). Moreover, on the one hand, two studies reported an increase in complex I subunits, ND1 and ND6, and a decrease in the complex III subunit, cytB, in comparison to normal controls. On the other hand, ET attenuated ND1 and ND6 expressions, and enhanced CytB levels (27, 33).

Regarding the end product of ETC, two studies reported a decrease in ATP production in IHD when compared with normal controls, while ET countered the effects of IHD and resulted in an increase in ATP production (24, 28). Nevertheless, the study by Kraljevic et al. only reported this increase in ATP production when using Complex I substrate (24).

#### Effects of ET on cardiac mitochondrial biogenesis in IHD

Eight studies (24, 25, 27, 28, 30, 31, 33) investigated the effect of ET on the cardiac mitochondrial biogenesis in IHD (Table 2 and Table 4A). Three studies reported enhanced PGC1α expression in IHD compared with the normal group (24, 27, 33), while the other three (3) studies reported differently (25, 26, 30). Nevertheless, five (5) studies reported enhanced PGC1α expression in the exercise-trained IHD compared with the sedentary control (25, 27, 30, 33), while the study by Kraljevic et al. (24) reported otherwise (24). Moreover, decreased expression of the mitochondrial biogenesis regulators AMPK (25, 28), NRF1 (25), TFAM (26), SIRT 1 (33), and SIRT 3 (25, 31) was reported in IHD in comparison to the normal controls, while ET enhanced the levels of these biomarkers. Meanwhile, one study reported an increase in the expression of NRF1 and TFAM (27) in comparison to normal controls, while two independent studies reported a decrease in NRF 1/2 (25) and TFAM (26). Nonetheless, all three studies reported that ET increased the expressions of these mitochondrial biogenesis biomarker (27).

Table 4Effects of ischemia and exercise training on cardiac mitochondrial quality control.

**Table:** 

**(A) Cardiac mitochondrial biogenesis References**	**Ischemic heart**										**Exercise training**									
	**PGC1α**	**PI3K**	**AKT**	**AMPK**	SIRT 1/ 3*	NRF 1/ 2*	**TFAM**	**POLG/PLOG**	**TOP1MTMT**	**SSBP1**	**PGC1α**	**PI3K**	**AKT**	**AMPK**	SIRT 1/ 3*	NRF 1/ 2*	**TFAM**	**POLG/PLOG**	**TOP1MTMT**	**SSBP1**
Kraljevic et al. (24)	↑										↓									
Jiang et al. (25)	↓	↓	↓	↓	↓*	↓*					↑	↑	↑	↑	↑*	↑*				
Jiang et al. (26)	↓						↓				↑						↑			
Tao et al. (27)	↑					↑/↑*	↑	↑	↑	↑	↑↑					↑/ ↑↑*	↑↑	↑↑	↑↑	↑↑
Lu et al. (28)		↑	↑	↓								↓	↓	↑						
Ebadi and Damirchi (30)	↓										↑									
Zhao et al. (31)					↓*										↑*					
Jia et al. (33)	↑	↑	↑		↓			↓	↓	↑	↑↑	↑↑	↑↑		↑			↑	↑	↑↑

PGC-1α, peroxisome proliferator-activated receptor gamma coactivator 1-Alpha; PI3K, phosphoinositide 3-kinase; AKT, protein kinase B; AMPK, AMP-activated protein kinase; SIRT3, sirtuin 3; NRF 1/2, nuclear respiratory factor 1/2; TFAM, mitochondrial transcription factor A; POLG, DNA polymerase gamma gene; TOP1MT, mitochondrial DNA topoisomerase I; SSBP1, single-stranded DNA binding protein; (Others) ↑, Increased; ↑↑, further increased; ↓, Decreased; Red Arrow, effect of ischemia; Blue Arrow, effect of exercise training.

**Table:** 

**(B) Cardiac mitochondrial dynamics and mitophagy**	**Ischemic heart**									**Exercise training**								
	**Mitochondrial**				**Mitophagy**					**Mitochondrial**				**Mitophagy**				
	**dynamics**									**dynamics**								
	**MFN1**	**MFN2**	**OPA1**	**DRP1**	**PINK**	**PARKIN**	**P62**	**LC3-II**	**BECLIN 1**	**MFN1**	**MFN2**	**OPA1**	**DRP1**	**PINK**	**PARKIN**	**P62**	**LC3-II**	**BECLIN 1**
Jiang et al. (26)	↓	↓	↓	↑						↑	↑	↑	↓					
Tao et al. (27)							↓	↑	↑							↑	↓	↓
Ghahremani et al. (29)	↑	↓		↑						↑↑	↑		↓					
Ebadi and Damirchi (30)		↓		↑		↓	↓				↑		↓		↑	*↑/↓		
Zhao et al. (31)	↑	↑	↑	↑	↓	↓	↑	↑		↓	↓	*↑↑/↓	↓	↑	↑	*↓/↑	↓	
Ghahremani et al. (32)									↑									↓

(Mitochondrial Dynamics) MFN1, mitofusin 1; MNF2, mitofusin 2; OPA1, optic atrophy 1; DRP1, dynamin-related protein 1. (Mitophagy) PINK, PTEN-induced kinase 1; P62, suquestosome 1; LC3II, microtubule-associated protein light chain 3. (Others) ↑, Increased; ↑↑, further increased; ↓, Decreased; Red Arrow, effect of ischemia; Blue Arrow, effect of exercise training; *, Refer to Table 1.

Upregulation in the intracellular signaling biomarkers PI3K and AKT in IHD compared with normal controls was reported in two studies (28, 33). This is in contrast with one study (25), which reported a decrease in the expression of the said biomarkers. The latter study reported an increase in the expression of these biomarkers in exercise-trained IHD, which was similarly reported by a recent study (33). On the contrary, one study reported that ET decreased PI3K and AKT (28).

One study reported an increase in the expression of the mitochondrial DNA (mtDNA) replication genes, *POLG/PLOG* and *TOP1MT*, in IHD in comparison to normal controls (27), while two (2) studies reported an increased SSBP1 expression (27, 33). On the contrary, ET upregulated the expression of these three mtDNA replication genes in both studies (27, 33).

#### Effects of ET on cardiac mitochondrial dynamics in IHD

Four studies (26, 29–31) analyzed the effect of ET on the cardiac mitochondrial dynamics in IHD (Tables 2, 4B). Looking into the effect of IHD and ET on the cardiac mitochondrial fission biomarker, DRP1, all four mentioned studies reported a downregulation of DRP1, whereas ET has reversed this effect (26, 29–31).

Different results on the fusion biomarkers were reported in the four studies. Of these, three (3) reported a decrease in MFN2 (26, 29–31), MFN1, and OPA1 (26) expressions in IHD when compared with normal controls, while two other studies reported an increase in the level of MFN1 (26, 31), MFN2, and OPA (31). ET caused upregulation in the expression of MFN 1 (26, 29), MFN2 (26, 29, 30), and OPA1 (26). On the contrary, one study (31) reported a decrease in MFN1 and MFN2 in exercise-trained IHD compared with sedentary IHD. In the similar study, contradicting results on the effect of ET in OPA1 were also reported.

#### Effects of ET on cardiac mitophagy in IHD

As part of the essential mechanisms involved in the mitochondrial quality control, four studies (27, 30–32) investigated the effect of IHD and ET on the mitophagy biomarkers (Tables 2, 4B).

A total of four independent studies, on the one hand, reported a decrease in the expression of the different mitophagy biomarkers, such as that of PINK (31), Parkin (30), and P62 (27, 30). However, one study reported an increase in P62 levels in IHD in comparison to normal controls (31). With ET, increased PINK (31) and Parkin (30, 32) levels were noted. The latter two studies, on the other hand, reported contrasting results on the effect of ET in P62, wherein both studies reported an increased and a decreased level of P62 in the exercise-trained IHD (30, 32).

Moreover, two (2) studies reported an increased LC3II expression (27, 31), while two other studies reported increased Beclin1 levels in IHD (27, 32) as compared to normal controls. On the contrary, ET reversed these downregulated autophagosomal biomarkers in IHD (27, 31, 32).

#### Effects of ET on cardiac mitochondrial characteristics in IHD

A total of four studies (25, 26, 31, 33) also analyzed the cardiac mitochondrial physiological characteristics in IHD, with and without ET (Tables 2, 3).

Apart from IHD, decreased mitochondrial density (25) and MMP (26, 33) were noted. Moreover, there was an increase in the number of mitochondria observed in IHD (33). ET regulated these effects of IHD, as manifested by an increase in cardiac mitochondrial density (25) and MMP (26, 33), and a decrease in cardiac mitochondrial number (33). Regarding the cardiac mitochondrial size, an opposite finding was noted. One study reported an increase in cardiac mitochondrial size in IHD compared with normal controls (31), while another study reported differently (33), whereas ET also caused reversal of each study's finding.

## Discussion

### Synthesis of evidence

**Figure 2:**
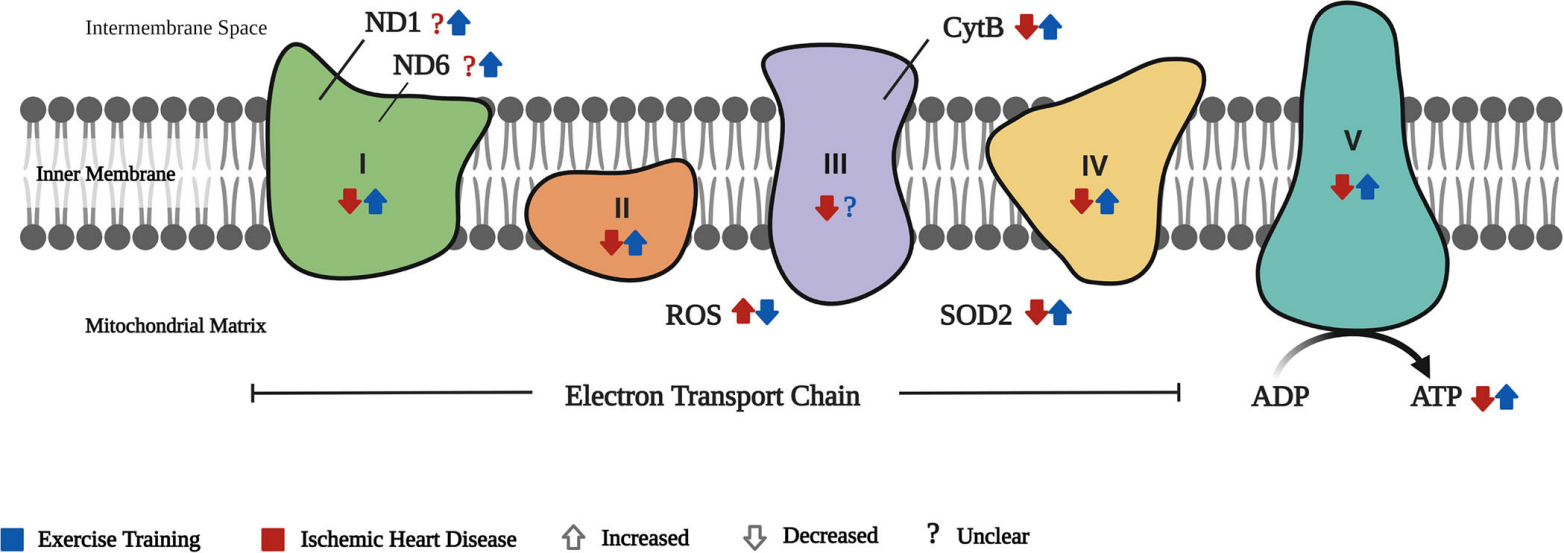
Cardiac mitochondrial respiration. The effect of IHD and exercise training (ET) on the cardiac mitochondrial respiration is depicted by the different components involved in the electron transport chain (ETC). ND1 and ND6 are components of the complex 1 of the ETC wherein there is no clear evidence of its effect in IHD, while ET increased the expression of these biomarkers. Complexes I–V are involved in the passage of electrons (with IHD–decreased; ET–increased complexes I, II, IV, and V, but no clear evidence on ET's effect in complex III). With IHD, ROS in the ETC is increased but is reversed by ET. Conversely, SOD2 is decreased in IHD and ET increases its expression. The end product of ETC, the ATP, is decreased in IHD, whereas ET improved its production.

**Figure 3:**
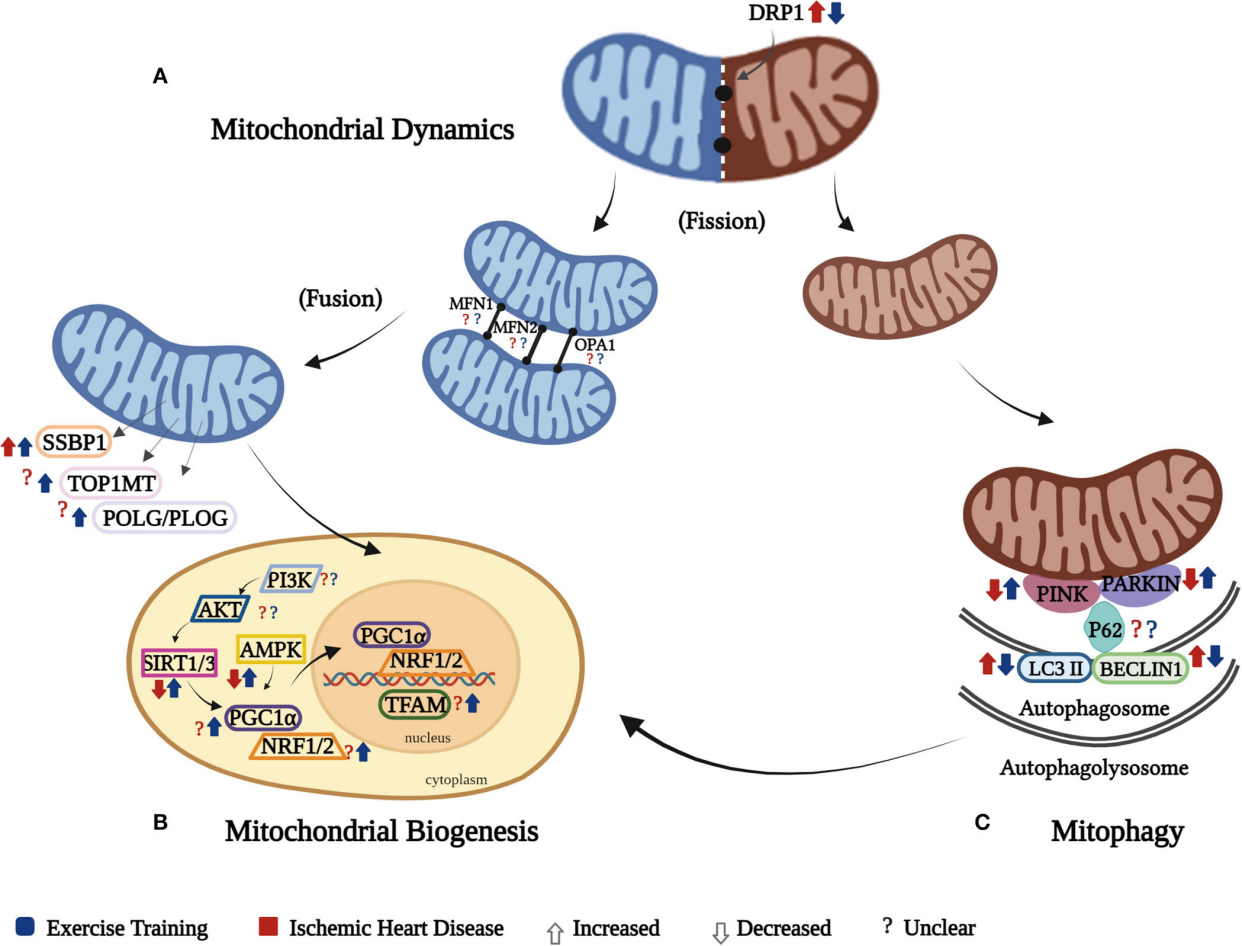
Cardiac mitochondrial quality control (MQC). The effect of IHD and ET on the cardiac MQC is presented separately through its three components: **(A)** the mitochondrial dynamics shows increased fission as a result of increased DRP1 in IHD and was reversed by ET, while there was an unclear effect of IHD and ET on cardiac mitochondrial fusion biomarkers (MFN1, MFN2, and OPA1); **(B)** mitochondrial biogenesis happens in response to the continuous need of energy, but in IHD,this mechanism is impaired as manifested by decreased transcription and translation processes due to diminished expression of SIRT3 and AMPK, which was reversed by ET, while both IHD and ET show unclear effects on AKT, PI3K, PGC1a, NRF1/2, POL/PLOG, TOP1MT. SSBP1, on the contrary, was increased in IHD and ET further increased it. Of note, PGC-1α is the key regulator in mitochondrial biogenesis, which might be triggered by SIRT1 or 3 and AMPK. The activated PGC-1α might not only enhance expression of the NRF1/2 to result in nuclear-encoded mitochondrial proteins, but also directly activate TFAM, which in turn to promote the transcription of mitochondria gene-encoded proteins in mitochondria; **(C)** mitophagy happens secondary to damaged mitochondria, which in IHD this mechanism is disrupted as manifested by decreased in PINK and Parkin, which were ameliorated by ET, while there is an unclear effect of both IHD and ET on P62. The autophagosomal factors, LC3II and Beclin 1, were increased in IHD, whereas ET attenuated their levels. As there is a continuous removal of mitochondria, and the process of mitophagy progresses on the autophagolysosomal level, it triggers the different mitochondrial biogenesis biomarkers to function in order to form new mitochondria. It should be noted that these three components work together, and affect the mitochondrial respiration.

The results of our systematic review show that ET improves the cardiac mitochondrial function as manifested by improved cardiac mitochondrial respiratory function through better and controlled expression of the different biomarkers involved in the ETC, ameliorating oxidative stress shown through decreased ROS, and increased SOD2 and ATP production. Enhanced cardiac MQC has also been proven as evidenced by controlled and/or improved expression of the different cardiac mitochondrial biogenesis, dynamics, and mitophagy biomarkers. In addition, an evidence of regulated cardiac mitochondrial characteristics was shown. These findings imply that ET can attenuate the damage caused by IHD in the heart by reversing the effects of the disease on cardiac mitochondrial respiration (Figure 2) and cardiac mitochondrial quality control (Figures 3A–C).

### Cardiac mitochondrial respiration

A sufficient and continuous supply of ATP is needed for the heart to function correctly. At least 60% of the ATP produced is usually utilized just for the contraction of the heart alone (34, 35). In IHD, however, the processes involved in ATP production are disrupted due to cardiac mitochondrial dysfunction. On the one hand, reports on weakened cardiac mitochondrial respiration are expressed through ETC complex activity deficits, a rise in ROS levels, and decrease in SOD2, RCR, and ATP production, leading to oxidative stress (3, 7). On the other hand, ET significantly restores the disrupted cardiac mitochondrial respiration.

Based on our findings, ET enhanced complexes I-V activity, RCR, and ATP production (24–26, 28). Supportively, different studies showed improved activity of the ETC complexes secondary to ET on both HF (36), and healthy young and old hearts compared with their sedentary counterparts (37).

Regarding ND1, ND6, and cytB, despite the difference in the type of ET used in the two included studies, both revealed an enhanced expression of these biomarkers (27, 33). It should be noted that swimming requires higher energy consumption than treadmill exercise due to the included properties of water to be dealt with while performing it. Therefore, greater cardiac stress can be expected. This was manifested by the increase in cardiac cross-sectional diameter reported in swim-trained diabetic rat hearts in comparison to those treadmill-trained rats (38). In connection to ND1, ND6, and cytB, one study on diabetic cardiomyopathy reported similar results of enhanced expressions of these biomarkers on treadmill-trained mice hearts (6). ND1 and ND6 of complex I, and CytB of complex III, are crucial for efficient mitochondrial activity, as these help in the electron transport for ATP production (39, 40). As these subunits partake in the ETC activity, and ET improves their expressions, we have assumed that ET can help induce better cardiac respiratory function in IHD.

In cardiovascular diseases, on the one hand, increased ROS production may lead to further disease progression. In IR, for example, ROS has a key role in IR-induced myocardial injury (41). On the other hand, SOD is the first line of defense to counter the effect of ROS (42). Hence, a balance between ROS and anti-oxidative enzymes (SODs) was essential (43), and this was proven by a previous study wherein increased ROS was balanced through the help of SODs (44). Our findings showed an imbalance in ROS and SOD2 expressions in IHD, which were regulated by ET (25, 31, 33). These findings are supported by previous reports of decreased ROS levels (45) and increased SOD2 production secondary to ET in IR (46) and IR-induced arrhythmia (47). As this SOD2 is found primarily in the mitochondrial matrix, it should be noted that enhanced SOD2 is crucial in the improvement of mitochondrial respiration, which helps induce cardioprotection (42). Moreover, as part of the cardiac mitochondrial respiration, the RCR is a practical measure in identifying mitochondrial dysfunction, as better RCR results are seen in more efficient ATP use and production (48). In IHD, our findings showed that both RCR and ATP were downregulated in IHD, and recovery on the expression of both RCR and ATP was evident secondary to ET (24, 26, 28). Similarly, two animal studies reported that a 5-day ET in IR (45), and an 8-week ET in HF (49) caused an increase in RCR. Regarding ATP, two other studies reported an increased ATP production in exercise-trained HF (49) and MI-induced hearts (50). Nevertheless, the latter only reported a comparison between MI and the exercise-trained MI; hence, there were no data showing its difference in normal hearts.

Previous studies reported the benefits noted in heart on mitochondrial ATP-sensitive potassium (mitoK_ATP_) channel opening *via* decreasing ROS production during precondition stage, and preventing disruption of the mitochondrial intermembrane space during IR (51, 52). Regarding the effects of ET, a previous study explained how ET contributes to sarcolemma ATP-sensitive potassium (sarcoK_ATP_) channel opening. This opening was believed to cause shortening of cardiac action potential, which in turn helps protect the heart from IR. Moreover, as sarcoK_ATP_ also contributes to mitoK_ATP_ opening, investigations have brought to the idea of how the latter also plays a role in cardioprotection (53). Identifying how ET affects the function of the mitoK_ATP_ channel could have then supported our findings. However, none of the included studies were able to investigate this matter. Therefore, it would be an interesting point of investigation in future studies.

### Cardiac mitochondrial quality control

The interplay of the different cardiac MQC mechanisms is essential to cardiac respiratory function. Concerning the cardiac mitochondrial biogenesis, it is established through the replication and transcription processes of various genes on both the mitochondria and nuclear levels (54). The downregulation of cardiac mitochondrial biogenesis in cardiac diseases is usually triggered by oxidative stress (8, 55). In our findings, however, inconsistent results on the effect of IHD on these cardiac mitochondrial biomarkers were noted (e.g., PGC1α, TFAM, POLG/PLOG, TOP1MT, PI3K, and AKT). These differences may have been attributed by the type of animal model and exercise utilized in the studies. Nevertheless, ET assisted in the increase and/or regulation of expression of SIRT 1/3, AMPK, PGC1α, NRF 2, TFAM, POLG/PLOG, TOP1MT, and SSBP1 (24–28, 30, 33).

The PGC1α plays a significant role on both mitochondrial respiration and mtDNA transcription and translation processes, as it controls the transcription of TFAM, which is also involved in the mtDNA maintenance (56, 57). In addition, NRF 1,2, and TFAM, which are the downstream transcription factors of PGC1α, together with the different mtDNA genes (*TOP1MT, SSBP1, POLG*/*PLOG*), play a significant role on both transcription and/or replication processes that help promote cardiac mitochondrial biogenesis (6, 58). Hence, the attenuation of PGC1α may lead to the disruption of the cardiac mitochondrial biogenesis. Despite some contradicting results we obtained, regarding what really transpire in the expression of PGC1α in the exercise-trained hearts, majority of the results are comparable to the study by Kemi et al., which reported that PGC1α expression was upregulated in the exercise-trained MI hearts in comparison to the sedentary controls (59). The factor that may have contributed to the difference in the result acquired by Kraljevic compared with other included studies determining PGC1α, however, is difficult to identify, as the study characteristics of these studies are somewhat similar, especially on the ET protocol administered. Regarding other mitochondrial biogenesis biomarkers, although the included studies in this review that investigated on TFAM and the identified mtDNA genes used a more extensive ET (swimming), previous studies reported similar results of increased levels of TFAM, TOP1MT, and SSBP1 in the treadmill-trained diabetic cardiomyopathy-induced hearts (6), and NRF2 in the treadmill-trained isoproterenol-induced hearts (60). This may imply that regardless of the type of ET, improvement of these mitochondrial biogenesis biomarkers can be expected. Nevertheless, the intensity and/or duration could be taken into consideration, especially that swimming is a more extensive type of ET.

PI3K and AKT are essential in IHD due to their role in preventing cardiomyocyte death secondary to oxygen deprivation. Through the activation PI3K/AKT signaling pathway, post-IHD cardioprotection can be established (61, 62). However, contradicting results regarding this issue were noted. These differences might have been influenced by the gender of the rat species used in the included studies (25, 28, 33). Nonetheless, two studies showed that ET in diabetic cardiomyopathy and hypertensive hearts increased the AKT phosphorylation (6, 63). In another study, a 10-week ET enhanced both AKT and PI3K levels in diabetic rat hearts (64). In IHD, dysfunctional mitochondria are typical; hence, the continuous interplay of cardiac MQC happens. However, extreme activation of mitophagy can lead to detrimental effects. This happening then is usually regulated by AMPK, through which its activation leads to mitochondrial metabolism (11, 65). Our findings showed that ET in IHD upregulated AMPK. In a similar study, Ma et al. reported that a 4-week swim training enhanced AMPK in isoproterenol-induced cardiac fibrosis and also caused its activation in AMPK-knockout mice (21).

Sirtuins 1 and 3 (SIRT 1/3) are the two most common sirtuins involved in cardiovascular diseases. Both sirtuins play an integral role in cardioprotection by regulating different mitochondrial functions concerning oxidative stress and/or cell death (66, 67). Dysregulation of any of these sirtuins may also lead to problems related to ATP production. Findings in this systematic review showed that ET recovered the downregulated levels of SIRT 1 and 3 in IHD (25, 31, 33). Our result is comparative with the study by Donniacuo et al. (68), which showed that MI-induced rat hearts enhanced the expression of SIRT 1 and 3 post-ET, which in turn caused cardioprotective mechanisms as manifested by attenuated cell death and oxidative stress (68). In a more recent study by Alavi et al. on dysfunctional rat hearts, ET was also noted to increase both SIRT 1 and 3 (69).

The four (4) included studies in this systematic review showed contrasting results about the cardiac mitochondrial fusion machinery (26, 29–31). Zhao et al. (31) presented decreased cardiac mitochondrial fusion after ET, which is in contrast to the results of the other three included studies. The differences in the results may be attributed to the differences in the animal model, and type of exercise used in the studies. The use of an aged animal model and swimming as a mode of ET, on which the latter was explained earlier requiring an extensive amount of energy in comparison to treadmill exercise, might have led to such differences in the results. Nevertheless, the other three included studies are supported by the investigations of Quiroga et al. and Ma et al. on hypertensive and hypertrophied hearts, respectively, which showed that an 8 to 16-week ET increased OPA1 levels (21, 63). In the study by Campos et al. (22), heart failure caused a significant increase in cardiac mitochondrial dynamics machinery (MFN1 and MFN2) compared with normal hearts, but ET upregulated the expression of both MFN1 and MFN2 (22). Our results that explained MFN 1 and MFN2 expressions in exercise-trained IHD yielded opposite results, which can be explained by the fact that heart failure causes more severe damage to the heart than IHD (MI or I/R). With this, the effect of ET in these biomarkers is dependent on the disease severity. Hence, to yield better results, given that mito-fusion plays an important role in the regulation of mitochondria, the type, duration, and/or intensity of ET to be provided should be in consideration to the severity of the disease.

The cardiac mitochondrial dynamics is essential in the renewal and degradation of damaged mitochondria through its fusion and fission mechanisms (70). In our findings, the increase in the cardiac mitochondrial fission biomarker (DRP1) secondary to IHD was reversed by ET, regardless of the type of ET used (26, 29–31). Supportively, one study showed that a 4- to 8-week ET revealed a decrease in DRP1 production in the exercise-trained transverse aortic constricted heart (21). Nevertheless, this downregulation of DRP1 should be balanced since continuous and deficient expression of this mitochondrial fission biomarker can also be detrimental in IHD.

A decrease in the expression of the cardiac mitophagy detectors, PINK (27), Parkin, and P62 (27, 30), were noted in this study, whereas ET enhanced their expressions. Interestingly, despite the differences in the type of ET used, the results of the two included studies reported a decrease in P62 expression in the lesser exercise intensity swim-trained (31) and treadmill-trained (30) MI hearts in comparison to their higher intensity-trained and sedentary counterparts. Nonetheless, this enhanced P62 expression in the higher intensity-trained hearts is comparable to the study by Tao et al. (27), which also used swimming as a form of ET. These results of increased P62 are also similar to a single-bout study of exercise-trained healthy rat hearts that has been compared with non-exercise trained hearts (71). With this, on the one hand, it can be assumed that P62 can be affected not specifically on the type of ET but on the duration and/or intensity at which it is administered; hence, the intensity and/or duration of ET to be provided among IHD patients must be taken into consideration. On the other hand, Li et al. reported an increased expression of PINK and Parkin in the exercise-trained MI-induced hearts (72). With these studies, it can be noted that ET aids in mitophagy activity. Nevertheless, despite these discoveries of increased PINK and Parkin levels in exercise-trained IHD, the lack of evidence on the effect of ET post-IHD on P62 makes it hard to speculate that ET promotes healthier cardiac mitochondria in IHD through the help of increased mitophagy biomarkers.

Furthermore, our findings show that autophagosomal factors (LC3II and Beclin 1) were decreased in exercise-trained IHD (27, 31). According to Wang et al., ET works in either of the following two ways: (1) it promotes autophagy in diseases causing decreased autophagy activity, or (2) it inhibits autophagy in diseases causing increased autophagy (73). Similar to our findings, an increase in LC3II expression was seen in MI-induced rat hearts, which was attenuated by a 4-week ET (74). Regarding Beclin 1 expression, Wang et al. mentioned that it is upregulated in IR-induced hearts, causing disease progression (73). Campos et al. otherwise reported that Beclin 1 expression is decreased in exercise-trained failing hearts (22). This evidence regarding the downregulation of autophagosomal factors in the cardiac mitochondria can be assumed to have been attributed by the excessive autophagy activity in IHD, which rescues the heart from further damage.

### Cardiac mitochondrial physiological characteristics

Our findings on the cardiac mitochondrial physiological characteristics showed a decrease in the number of cardiac mitochondria produced and an increase in the mitochondrial density, shape, and MMP (25, 26, 31, 33). In addition, there were opposite results from this review concerning the cardiac mitochondrial size. Zhao et al. (31) reported a decrease in the cardiac mitochondrial size, while Jia et al. (33) reported otherwise. Nonetheless, regarding the cardiac mitochondrial number and size, one study (22) showed similar results with the study by Jia et al. (33), where ET decreased cardiac mitochondrial number with increased sizes in HF. As mentioned in the earlier discussion, cardiac mitochondrial dynamics involve fusion and fission mechanisms. With ET, cardiac mitochondrial number and size are regulated through proper mitochondrial dynamic mechanisms. Given this, we can assume that this may have caused the regulation in the number and size of mitochondria. Regarding MMP, Zorova et al. (75) mentioned that the electrochemical potential of the different ions present in the mitochondrial membrane assists in regulating ATP synthesis (75). This then shows the importance of MMP in the structure and metabolism of cardiac mitochondria leading to better cardiac mitochondrial function (75). As our findings show that ET ameliorated the decreased in MMP (26, 33), the result may then signify a better cardiac mitochondrial function.

### Limitations of the study

Some limitations were identified in this systematic review. First, we only concentrated on English-written articles searched from three (3) databases (PubMed, EMBASE, and Web of Science), resulting in including only ten (10) articles in this systematic review. Second, we only considered articles that used acute-IHD (MI and IR) models; hence, the articles that used HF-induced models were excluded despite it being the consequence of prolonged IHD, limiting our review in identifying to what extent ET can be conducted in cardiac injury to facilitate cardioprotection. Third, we only included articles with treatment duration of ≥ 3 weeks, which limited our study in reporting the immediate effect of ET post-IHD. Lastly, our study did not associate treatment protocol (intensity, duration, and frequency) with both the cardiac mitochondrial respiration and the cardiac MQC, making it challenging to identify the best treatment protocol for IHD.

### Clinical implications

The cardioprotective effect of ET on IHD is presented in this systematic review through improved cardiac mitochondrial function. This is evidenced by different studies that explored on how ET can reconstruct the cardiac mitochondria that has been dysregulated by different heart ailments such as IHD and HF (22, 76). Moreover, different studies also showed how ET improves cardiorespiratory endurance and different cardiovascular risk factors, which in turn helps improve the QoL of IHD patients (77, 78).

Nevertheless, further studies regarding this matter are encouraged using an improved methodology, such as utilizing human subjects or having pre- and post-evaluation results on the same subjects (human/animal), while also identifying the cardiorespiratory capacity (e.g., VO_2_ max). Maximum oxygen uptake (VO_2_ max) has been observed as a valid and important measurement in determining the extent of the cardiopulmonary system (79). Through adding information about VO_2_ in such studies about cardiac mitochondrial function in IHD, improved methodology can be adhered, which may help provide a more extensive understanding on the effect of ET in IHD. Moreover, it is also suggested to investigate the correlation between ET protocol (intensity, frequency, and duration) and the cardiac mitochondrial function in order to identify the most efficient exercise protocol for IHD subjects.

## Conclusion

Based on the results of our systematic review, it was observed that ET was able to counteract the effects of IHD, as it: (1) ameliorated the cardiac mitochondrial respiratory function; (2) restored the cardiac mitochondrial quality control mechanisms as manifested by improved cardiac mitochondrial biogenesis, cardiac mitochondrial dynamics, and cardiac mitophagy; and (3) mended the disrupted cardiac mitochondrial physiological characteristics.

With all these points, we conclude that ET helps control cardiac cell death by improving cardiac mitochondrial function (respiration and MQC). Hence, ET is a suitable medium for cardioprotection in IHD.

## Data availability statement

The original contributions presented in the study are included in the article/supplementary material, further inquiries can be directed to the corresponding authors.

## Author contributions

MADV, QL, and S-DL contributed to the conceptualization. NTN, Y-JC, YL, and Z-YC contributed to the methodology. MADV, WL, NTN, Y-JC, YL, and Z-YC contributed to the collection, synthesis, validation, and interpretation of data. MADV drafted the manuscript. NTN, QL, WL, and S-DL edited and revised the manuscript. All authors approved the final version of the manuscript.

## Funding

This study was partially supported by Weifang Medical University, China Medical University, and the project (Grant no. 201940388) from Shanghai Health Committee.

## Conflict of interest

The authors declare that the research was conducted in the absence of any commercial or financial relationships that could be construed as a potential conflict of interest.

## Publisher's note

All claims expressed in this article are solely those of the authors and do not necessarily represent those of their affiliated organizations, or those of the publisher, the editors and the reviewers. Any product that may be evaluated in this article, or claim that may be made by its manufacturer, is not guaranteed or endorsed by the publisher.
